# Deformation of the Internal Connection of Narrow Implants after Insertion in Dense Bone: An in Vitro Study

**DOI:** 10.3390/ma12111833

**Published:** 2019-06-06

**Authors:** Rafael Delgado-Ruiz, Ana Nicolas Silvente, Georgios Romanos

**Affiliations:** 1 Prosthodontics and Digital Technology, School of Dental Medicine, Stony Brook University, Stony Brook, NY 11794-8712, USA; 2 Restorative Dentistry, School of Dentistry, Murcia University, 30008 Murcia, Spain; ainicolas@um.es; 3 Periodontology, School of Dental Medicine, Stony Brook University, Stony Brook, NY 11794-8712, USA; georgios.romanos@stonybrookmedicine.edu

**Keywords:** narrow dental implants, internal connection, deformation, dense bone

## Abstract

Implant connections must resist surgical and prosthetic procedures without deformation. This study evaluated the deformation of different internal connections (IC) of narrow dental implants (NDI) after their insertion in artificial dense bone. Thirty NDI, with different IC geometries, Group A (internal hexagon), Group B (tri-channeled), and Group C (four-channeled), with the same length and similar narrow diameters, were inserted in type II density bone blocks. Drilling protocols for dense bone from each implant manufacturer were followed. The Insertion torque (IT), connection length, vertex angles, and wall deformations were analyzed before and after the insertion of the implants. ANOVA (Analysis of Variance) and Tukey post-test were used for statistical comparisons. IT values were higher for Group A, surface damage, and titanium particles were observed in the IC in all the groups. Angle deformations between 5 and 70 degrees were present in all the groups, and the walls of Group B connection were the most affected by deformations (*p* < 0.05). Within the limitations of this experiment, it can be concluded that narrow diameter implants will suffer deformation of the implant connection and will also experience surface damage and titanium particle release when inserted in type II bone density.

## 1. Introduction

Two-piece dental implant systems include an intraosseous part (the implant body) and an extraosseous component (the prosthetic abutment) that are usually connected with a retention/prosthetic screw [1]. The union between these two parts occurs at the implant–abutment interface (IAI) [2], also known as the implant–abutment junction (IAJ) or the implant–abutment connection (IAC) [3].

For single-implant restorations, the union of the implant and the abutment must be stable enough to resist functional loads and to reduce screw loosening [4,5]. Different geometric designs have been introduced for the implant connection (IC) and the prosthetic abutment connection (AC) with the purpose of facilitating the implant’s insertion, achieving a proper record of the implant’s orientation during the impression, improving the mechanical engagement between parts at the IAI, enhancing the mechanical strength, and reducing the screw loosening [6].

This geometric feature is called the “implant/prosthetic index”, and is defined as “a core or mold used to record or maintain the relative position of dental implants or teeth, to a cast, or to some other structure” [7]. The implant index corresponds, in terms of design, with its counterpart, the prosthetic abutment index, and can be located inside the implant’s body below the level of the implant platform (internal connection) or above the level of the implant platform (external connection) [7].

The existence of angles, straight walls, channels, boxes, tubes, and cones in the connection’s design also prevents rotation between the components of the system [8,9]. The structural integrity of these geometric features at the level of the IC is crucial for the long-term stability of the IAI and the survival of the prosthetic restoration [10]. A damaged prosthetic abutment can be replaced; however, the deformation of the IC cannot be reversed, and the implant may become non-restorable [10].

Different factors can induce deformation of the implant connections while the implant functions, including overload, over-torqueing, and non-axial loads [11], a non-passive fit of the restoration and forced prosthetic screw tightening [12,13], narrow implant diameters [14], the presence of a micro-gap and micro movement between the parts [15], frictional wear [16], multiple connections and disconnections of the prosthetic abutment [17], and the thickness of the implant walls [18,19].

Recently, the action of implant insertion has been described as another potential factor for deformation of the IC [20]. The insertion torque forces produced during the implant’s insertion are transferred to the implant index of the IC, causing its potential deformation when the applied force exceeds the modulus of elasticity [21]. Apparently, external connections suffer greater deformation than internal connections [22]. Although less deformation might be experienced by internal connections compared to external connections, internal connections can also be deformed during the implant’s insertion, which can result in potential enlargement of the micro-gap and, thus, increased wear and bacterial leakage [23]. To date, the magnitude of the deformation of the IC of narrow dental implants has not been evaluated, and there is a lack of information about the deformation pattern of different IC geometries of narrow implants after their insertion in Type II bone.

Therefore, the goals of the present study were to evaluate the insertion torque, the amount of deformation, and the characteristic pattern of distortion experienced by narrow dental implants with three different types of IC geometries after their insertion in artificial Type II bone.

## 2. Materials and Methods

### 2.1. Bone Samples

Artificial bone blocks (Sawbones, Pacific Research Laboratories, Vashon, CA, USA) with a polyurethane foam structure and a density of 40 pounds per cubic foot (PCF) (0.64 g/cm^3^), resembling Type II bone, were fixed in a vise. These blocks are used as standard analogs for the evaluation of biomechanical properties of fixation plates, fixation screws, and dental implants [24]. A graphite pencil was used to mark the future implant beds at intervals of 10 mm. Thirty marks were made in each block.

### 2.2. Dental Implants and Experimental Groups

The IC of three different titanium dental implants, each with a tapered body and being of the same length and a similar narrow diameter, was evaluated (Table 1).

#### 2.2.1. Group A (Internal Hexagonal Connection)

Group A contained 10 Nobel Replace^®^ (Nobel Biocare, Goteborg, Sweden) conical connection dental implants, with a narrow platform (3.5 mm in diameter) and a length of 10 mm. These implants are characterized by a connection with six straight walls that form a hexagon as an anti-rotational feature.

#### 2.2.2. Group B (Internal Three-Channel Connection)

Group B contained 10 Nobel Replace^®^ (Nobel Biocare, Goteborg, Sweden) Trilobe^®^ (Nobel Biocare, Goteborg, Sweden) connection dental implants, with a narrow platform (3.5 mm in diameter) and a length of 10 mm. These implants are characterized by a three-channel/lobe connection with curved walls with convexities and concavities and rounded angles as anti-rotational features.

#### 2.2.3. Group C (Internal Four-Channel Connection)

Group C contained 10 Bone Level^®^ (Straumann, Basel, Switzerland) CrossFit^®^ connection dental implants, with a narrow platform (3.3 mm in diameter) and a length of 10 mm. These implants are characterized by connections with channels and boxes as anti-rotational features.

### 2.3. Standardization of the Implant Insertion

A single operator was calibrated for the drilling and insertion of narrow dental implants with three different internal connection designs. The operator followed each manufacturer’s recommended drilling protocol for the insertion of tapered implants in artificial Type II bone blocks.

For the standardization of the implant insertion, 10 implant beds were prepared, and the implant insertion was repeated 10 times per group. The implant platform was left at the level of the bone block’s surface. The intraclass coefficient (ICC) for the implant insertion level was used as a calibration reference. Zero millimeters of difference between the implant platform and the bone level was considered to be 100% agreement; <0.5 mm of difference between the implant platform and the bone level was considered to be 90% to 99% agreement; 0.5 to 1.0 mm of difference between the implant platform and the bone level was considered to be 90% to 80% agreement, and 1.0 to 1.5 mm of difference between the implant platform and the bone level was considered to be 80% to 70% agreement. A value equal to or higher than 80% was considered to be a reliable ICC.

### 2.4. Experimental Procedures

The drilling sequences for dense bone (Type II bone) were completed as follows:

- Group A drilling sequence

A pilot drill with a 2.0 mm diameter followed by a stepped drill with a 2.4/2.8 mm diameter, a stepped drill with a 2.8/3.2 mm diameter, and a screw tap with a 3.5 mm diameter. The drilling depth was 10 mm for all of the drills.

- Group B drilling sequence

A pilot drill with a 2.0 mm diameter followed by a conical drill with a 3.5 mm diameter and a bone tapping drill with a 3.5 mm diameter. The drilling depth was 10 mm for all of the drills.

- Group C drilling sequence

A pilot drill with a 2.2 mm diameter followed by a bone level tapered (BLT) drill with a 2.8 mm diameter, a BLT drill with a 3.3 mm diameter, and a bone tap drill with a 3.3 mm diameter. The drilling depth was 10 mm for all of the drills.

#### 2.4.1. Evaluation of the Insertion Torque 

The implants were inserted using an implant driver (Group A and Group B) or by mounting a pre-mounted implant (Group C). For the implants inserted using an implant driver, each implant driver was replaced after five uses.

The insertion torque (IT) values achieved when the implants were inserted at a depth of 10 mm into the bone blocks, and the implant platforms were flush with the bone block surface, were registered and are expressed in Newton centimeters. Ten IT values were recorded for each group, and the mean and standard deviations were obtained.

#### 2.4.2. Evaluation of the Implant’s Geometry and Its Deformation

The changes at the implant connection and the changes in the index geometry were evaluated for all of the implants included in this experimental study using a last-generation three-dimensional (3D) digital microscope (Keyence VHX-6000, Keyence Corporation, Osaka, Japan). The surface changes that each connection suffered after insertion into the dense bone were recorded. A description of the changes at the implant platform is provided, and the apparent deformation of the walls and implant index is listed.

A magnification of 50× was used for the measurement of the index geometry. For the evaluation of the connection length, the microscope was focused on the deepest portion of each connection using a magnification of 100×. For the qualitative evaluation of the IC characteristics, a magnification of 150× was used. 

The implants were positioned in the center of the microscope’s stage using an implant holder in the vertical position. A screen cross allowed us to reproduce this axial centered position for each implant. Images of the IC before and after insertion were obtained for all of the implants.

#### 2.4.3. Measurement Procedure

Once the deepest part of the IC had been located and focused upon, the motorized stage was programmed for vertical scanning with a step displacement of 5 µm (down–up). The vertical displacement was regarded as complete once the most coronal external part of the connection was in focus; thus, the whole IC was captured by this procedure. The total scanned length was dependent on the connection depth.

The VHX-6000-950F measurement data software (Version 2016, Keyence Corporation, Osaka, Japan) was used to register the landmarks and to complete the measurements. The following measurements were obtained before and after each implant’s insertion:

Connection depth: The distance from the bottom of the IC to the surface of the implant platform was measured. To obtain the mean IC length for each implant group, the arithmetic mean and standard deviation of the IC length of the 10 implants in each group were calculated and are expressed in millimeters.

Hexagonal connection angle: The angles that formed between the walls of the internal hexagonal connections were obtained. Six angles were obtained per implant; thus, 60 angles were obtained in this group.

**Figure 1:**
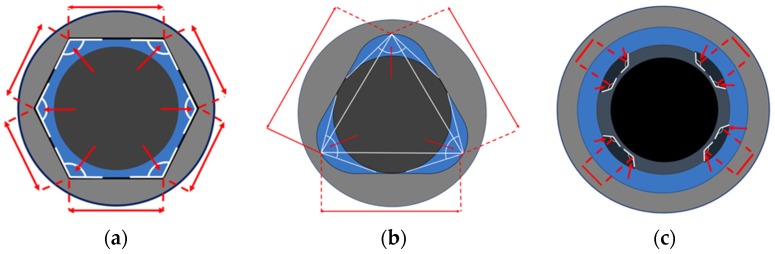
(**a**) Variables evaluated in Group A (Internal hexagon). The angles formed between the sides of the hexagon were located and measured. The length of the hexagon sides was also measured to determine changes at the internal connections (IC) geometry. (**b**) Variables evaluated in Group B (Internal Three-Channeled). The lobe angle was located with the vertex at the most convex portion of the channel and the sides at the base of the channel walls. The length of the wall was estimated by connecting the vertex of each channel to create a triangle. Each side of the triangle was considered for the evaluations. (**c**) Variables evaluated in Group C (Internal Four-Channeled). The anti-rotational features are small boxes protruding from the IC walls to the center of the implant connection. The internal angles of the boxes were measured. The length of the inner wall of each box was measured.

Hexagonal connection length of the wall: The central points at each vertex of the angles were located and connected; this resulted in six straight lines. The arithmetic mean and standard deviation of the length of the six lines that were obtained per implant were collated with the other implants inside the group. Sixty measurements were obtained in this group, and are expressed in microns as the mean and standard deviation (Figure 1a).

Three-channel connection angle: The angles formed between the vertex of the lobe (channel) and its side walls were recorded. The median and interquartile range of the measurements of three angles per implant were registered; thus, 30 measurements were obtained in this group. (Figure 1b). Three-channel connection length of the wall: The vertex of the three lobes was located and connected with a straight line. Three measurements were performed per implant to obtain the length of the wall. Thirty values were obtained per group; these values are expressed in microns. The mean and standard deviation were registered (Figure 1b).

Four-channel connection angle: The internal boxes of the connection were evaluated by measuring the angle that formed between each box’s walls. Four angles were obtained per implant; thus, 40 measurements were obtained in this group. The median and interquartile range of the angle measurements were calculated. Four-channel connection length of the wall: The distance between the innermost walls of the boxes, facing the center of the connection space, was measured and is expressed in microns. Four measurements were obtained per implant; thus, 40 measurements were obtained within this group. The mean and standard deviation were registered (Figure 1c).

### 2.5. Statistical Analysis

For the variables IT, connection depth, and linear distance (the length of the walls for all groups), an analysis of variance (ANOVA) with Tukey’s post hoc test were used to determine differences between groups. For the angle measurements, the median and the interquartile range were calculated, and non-parametric Kruskal–Wallis statistical analyses were performed to determine if the median values were different before and after insertion. Statistical significance was set at *p* < 0.05.

## 3. Results

The operator calibration showed an intraclass coefficient of 0.95 (less than 0.3 mm of difference) and was considered highly reliable. No single narrow implant fractured during the insertion process.

### 3.1. Qualitative Findings

All of the analyzed IC designs suffered surface alterations after insertion in dense bone. However, each group expressed different patterns of deformation.

#### 3.1.1. Group A (IC Hexagon)

**Figure 2:**
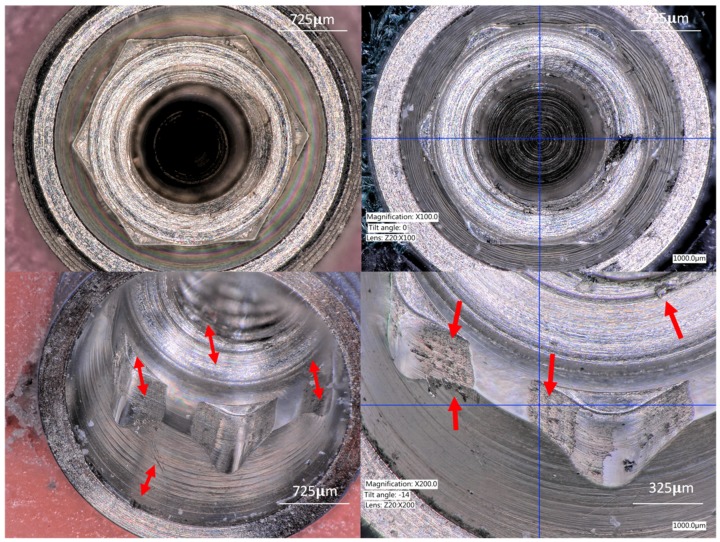
Upper left image shows a coronal view of Group A (Internal hexagon) Upper right image shows a coronal view of the same group with some scratches and widening of the vertex angles. Lower left image shows scratches at the internal cone and areas of deformation indicated by the red arrows. Lower right image is showing surface damage and titanium particles at the edges of the connection walls released during the implant insertion.

The internal walls showed titanium delamination and scratches. Titanium particle debri was observed at the external edges of the connection and at the base of the connection. In addition, the implant platform showed scratches, and the edges of the hexagon walls showed blunting in some areas. The hexagon angles appeared to be wider (Figure 2).

#### 3.1.2. Group B (Three-Channel)

**Figure 3:**
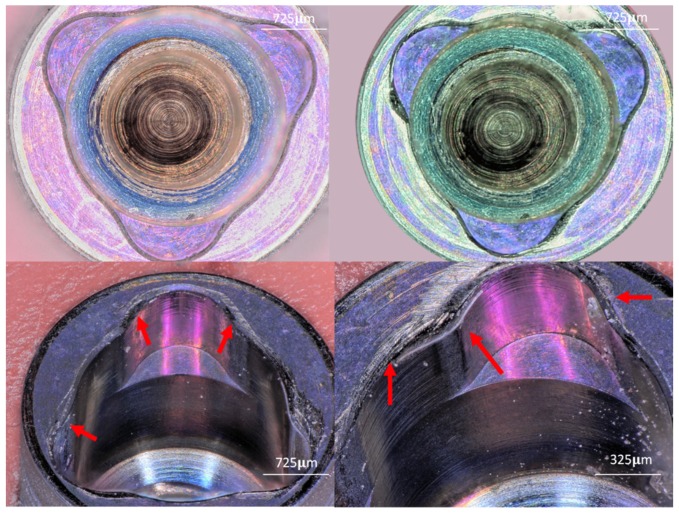
Upper left image shows a coronal view of Group B (Internal Three-Channeled). Upper right image shows a coronal view after implant insertion, the color-coded area is missing in some areas, and deformation of the channel wall is evident. Lower left image shows a close view of Group B after the insertion. The internal geometry is altered, deformation of the internal walls of the channel indicated by red arrows. Lower right image shows titanium particles at the implant platform and implant walls.

The platform of the implant showed scratches and loss of the color code that is used to identify the implant’s diameter. The walls of this IC showed scratches and compressive deformation at the edges of the channel’s base. Titanium particles were observed in different areas (Figure 3).

#### 3.1.3. Group C (Four-Channel)

**Figure 4:**
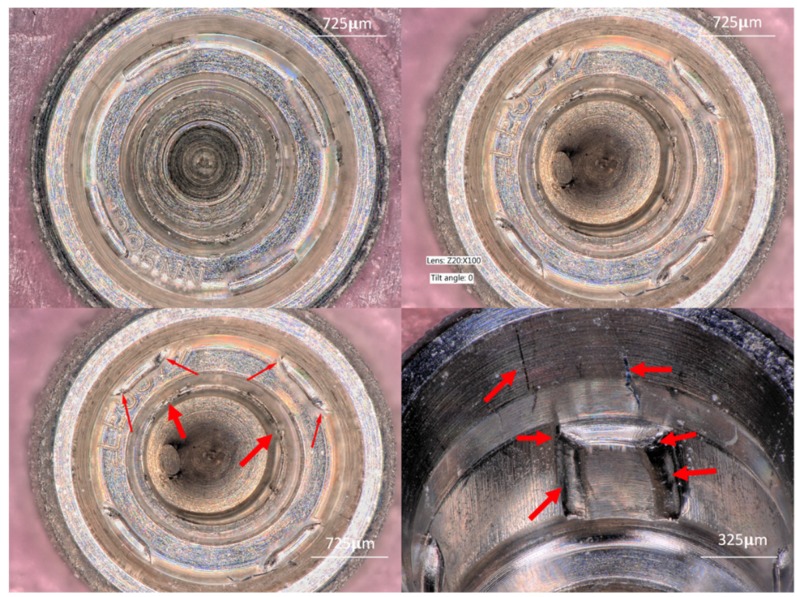
Upper left image shows a Group C (Internal Four-Channeled) narrow implant. The anti-rotational internal boxes, with their lateral walls forming a straight angle with the implant walls. Upper right image shows the deformation of the channel walls. These were deformed after insertion; the sides of the small boxes suffered plastic deformation and were flattened. Lower left image shows red arrows demonstrating titanium particles and deformation of all four channel elements. Lower right image shows the box walls completely deformed, the edges of the wall contained titanium particles. In addition, some scratches are observed.

The walls showed scratches, blunting, and deformation at the edges of the anti-rotational boxes. Furthermore, titanium particle accumulation was observed at the base of the boxes, and some scratches were observed at the internal cone (Figure 4).

### 3.2. Quantitative Findings

#### 3.2.1. IT values

The highest insertion torque values were obtained for Group A (37.5 ± 1.5 Ncm), followed by Group C (35.0 ± 2 Ncm) and Group B (33.5 ± 1.5 Ncm) (*p* < 0.05).

#### 3.2.2. Connection Depth

**Figure 5:**
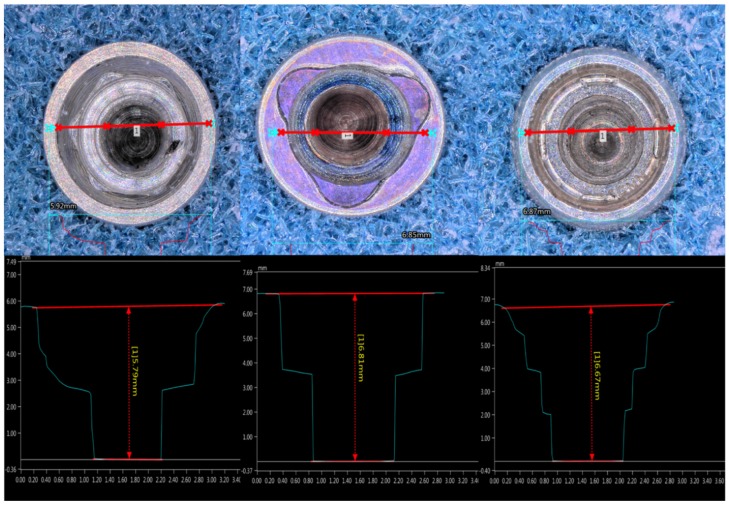
Length of the implant connection. Two points were located at each side of the implant platform. A line was traced from the most upper part of the platform to the lowest part of the connection. The blue lines are showing the internal profile of each connection, which is different in each of them. Group B has the longest connection depth.

Group B showed the largest connection depth (6.81 mm ± 0.13 mm), followed by Group C (6.67 mm ± 0.09 mm) and Group A (5.79 mm ± 0.1 mm). Group A was found to possess the shortest of the connections (by ±1 mm). There were no changes in the IC’s depth after the implant’s insertion in any of the groups (Figure 5).

### 3.3. Geometry Changes

#### 3.3.1. Group A (IC Hexagon)

Vertex angle: After the implant’s insertion, the vertex angle increased from a median angle of 119.29 to a median angle of 120.755. The Kruskal–Wallis test showed no significant difference between medians before and after insertion.

Length of the wall: There were minimal changes in the length of the walls after the implant’s insertion. The initial mean length was 1005.52 µm ± 2.11 µm; after insertion, the mean length was 1005.77 µm ± 7.4 µm. The ANOVA test showed a difference of 25.2 µm after insertion, and the Tukey’s test showed no statistically significant differences between means (*p* = 0.9658).

#### 3.3.2. Group B (IC Three-Channel)

Internal Lobe angle: After the implant’s insertion, the median value of the internal lobe angle increased from 95.25 to 97.975. The Kruskal–Wallis test showed no significant differences before and after insertion.

Length of the wall: After the implant’s insertion, there were minor variations in the length of the walls. The initial mean length was 2646.90 µm ± 4.21 µm; after insertion, the mean length was 2624.14 µm ± 27.6 µm. The ANOVA test showed a difference of 20.077 µm after insertion, and the Tukey’s post hoc test showed no statistically significant differences when comparing the length of the wall before and after insertion (*p* = 0.3878). 

#### 3.3.3. Group C (IC Four-Channel)

Internal box wall angle: The walls of the boxes of the IC were deformed after insertion. The median value of the angle before insertion was 92.025; after insertion, the median angle increased to 142.233. The Kruskal–Wallis test showed a significant difference between the median of the internal box angle before insertion and the median of the internal box angle after insertion.

Inner wall box length: After insertion, all of the inner walls were shortened. The initial mean length was 655.89 µm ± 4.21 µm; after insertion, the mean length was 469.68 µm ± 27.6 µm. The ANOVA test showed a difference of 186.21 µm, and the Tukey’s post hoc test showed a statistically significant difference between groups (*p* = 0.0006) (Table 2 and Table 3).

## 4. Discussion

This experimental study aimed to evaluate if narrow dental implants with different IC geometries suffered deformation after their insertion into Type II artificial bone. The insertion torque, the magnitude of the deformations, and the characteristics of the deformations were evaluated. The last-generation digital microscope allowed for the non-destructive 3D evaluation of the implant index and a non-distorted representation of potential changes in the implant index. In addition, the variation that may arise from one sample to another was excluded because the system was calibrated to a NIST-traceable standard. The drilling protocol recommended by each manufacturer for Type II bone was strictly followed. Thus, a comparable simulated clinical situation was achieved for all the experimental groups. Insertion torque values were obtained for each group to identify whether there was a relation between the degree of damage and the insertion torque values. The IT values obtained in the present work were all less than 37 Ncm. Group A achieved higher IT values, which were potentially produced by two factors: the thread design and substantial discrepancies between the final drill and the implant geometry compared to the other implant groups.

The IT values required to insert the implants into Type II bone were sufficient to produce visible wear and titanium particle delamination on all of the evaluated IC designs. Besides this, the implant index changed in all the implants. These results are in agreement with Kwon et al., 2009 [21], who evaluated changes in the rotational freedom of an implant connection after the application of insertion torque with different values (45 Ncm and 100 Ncm). The authors observed increased rotational freedom with 45 Ncm; however, they did not evaluate the amount of deformation. In contrast, in the studies performed by Teixeira et al., 2015 [10] and Nary Filho et al., 2015 [25], the authors observed standard implant deformation after the application of higher torques. Indeed, Teixeira et al., 2015 [10] performed torsion tests on external hexagon, internal hexagon, and Morse taper connections using torque values of 80 Ncm and 120 Ncm. They observed that both torque values produced deformation of the connections; the deformation was higher in the external connection implants and lower in the implants with the Morse taper connection. Nary Filho et al., 2015 [25] evaluated the rotational failure of Morse cone and external hexagon connections. The authors found rotational failure with torque values starting from 2.69 Nm (269 Ncm). These differences could be produced based on the diameter of the implants used in these experiments (>4.0 mm) [10,14], and their different composition (Ti–Al–V alloy) which possesses higher elasticity moduli [26].

In the present work, the evaluation of the IC characteristics after the implant’s insertion into Type II bone showed titanium particle delamination and accumulation of titanium particles in different areas inside all of the connections. Although the weight/percentage of titanium particles released from the IC during the implant’s insertion was not quantified in this study, this finding was made in all of the groups independent of the IT value required for the implant’s insertion. These results support previous systematic reviews that mention implant insertion as one of the causes of the release of titanium particles in implant dentistry [27,28]. We hypothesize that these particles originate from the IC (based on the surface changes and deformations). However, they may also originate from the implant driver, the implant mounting (the components of the interface that are subject to torsional forces) and from residues from the artificial bone used in this experiment.

The angles of the connections were evaluated to understand if the IT forces could bend or deform the implant index of narrow implants when inserted into Type II bone. The results of the present work demonstrate that the IT required to insert the implants produced a slight deformation of the vertex angles of the hexagon and tri-channel connections. Besides this, the four-channel connections demonstrated the highest deformation and a flattening of the internal box angles. Apparently, in narrow-diameter implants, a higher probability of failure could be expected, as described by Watanabe et al., 2015 [29], who showed that narrow-diameter implants (3.3 mm and 3.8 mm in diameter) possess a lower fracture torque and yield strength than wider-diameter implants (4.3 mm and 6 mm in diameter).

Two of the experimental groups in the present study used an implant driver for the insertion of the implants (Group A and Group B), and the other group (Group C) used a pre-mounted implant. It is believed that the implant mounting protects the implant index when high IT values are produced during the implant’s insertion. Thus, the forces are transferred to the mounting, causing its deformation/failure but maintaining the integrity of the implant index [21,30]. However, even the members of Group C, where the implants were inserted using the mounting, suffered deformation of the implant index. We hypothesize that the tested narrow implants suffer deformation at the IC with IT values between 33 Ncm and 37 Ncm. A pre-mounting system can be used to reduce the damage transferred to the IC, but cannot be used to avoid damage altogether.

The length of the connection’s walls was evaluated to identify whether the insertion of the implants in Type II bone might produce a large amount of deformation in the IC geometries. None of the evaluated groups showed significant changes in the length of the walls. This means that all of the forces applied during the implant’s insertion were transferred as deformations to the anti-rotational elements but not to the external walls of the implant’s body. This can be explained based on the IT values that were within the elastic modulus of the evaluated implants [31].

The present study possesses some limitations. First, only three internal connection designs were tested, which might exclude other current internal connections used for narrow implants. Second, wider implant diameters with the same connection design may present a different behavior, and therefore, other implant diameters with the same connections should be evaluated. Third, the connection geometries of the evaluated groups were different; therefore, different specific landmarks were evaluated for each group. Thus, the presented deformations show the behavior within each group, but comparisons between the groups cannot be performed. Therefore, to improve the extrapolation of data, the same geometries should be compared. Lastly, chemical evaluation should be included to confirm the elemental composition of the fragments and particles observed inside the implant connections to eliminate the risk of data misinterpretation. 

The results of the present investigation should be considered for the implants and connections evaluated only. It is probable that dental implants manufactured with titanium alloys and other connection designs present different behaviors. Therefore, further evaluation is recommended to confirm the findings of the present work with different implant connections, implant diameters, and alternative implant materials and titanium alloys.

The strengths of the present work lie in the calibration of the operator, the strict experimental protocol utilized, which increases the reliability of the obtained data, the non-destructive technology utilized for the evaluation of the IC geometry and deformation, and the selection of a few experimental variables that allow for the use of a robust statistical comparison.

This initial deformation of the IC produced during the implant’s insertion might be responsible for an increase in micro-gap/misfit dimensions, micro movement, screw loosening, and titanium particle release.

## 5. Conclusions

Narrow-diameter implants with 3.3 mm and 3.5 mm diameter and different internal connection designs (hexagon, three-channeled, and four-channeled) will suffer different levels of deformation and will experience surface damage and titanium particle release when inserted into Type II (dense) bone. The clinical relevance of the present work is that correct implant handling and proper implant bed preparation are essential to a reduction in deformation and the release of titanium particles in the implant index during the insertion of narrow implants in Type II bone.

## Figures and Tables

**Table 1:** Characteristics of the three narrow implant designs used for this in vitro experiment. Implant geometry, dimensions, characteristics of the connection, and composition are provided.

Connection Characteristics	Implant Manufacturer/Line	Coupling System	Implant Index Geometry	Composition	Insertion Device	External Geometry	Diameter	Length
**Group A** **Straight walls** **n = 10**	Nobel Biocare Nobel Active	Conical Friction fit Angle 12°	Internal Hexagon Six abutment positions	Cold worked Titanium Grade IV	Implant Driver	Tapered	3.5 mm	10 mm
**Group B** **Channeled walls** **n = 10**	Nobel Biocare Nobel Replace	Butt Joint Angle 0°	Three channels Three abutment positions				3.5 mm	
**Group C** **Channeled walls** **n = 10**	Straumann Bone Level	CrossFit Conical Friction fit Angle 15°	Four channels Four abutment positions		Pre-mounted/Transfer		3.3 mm	

**Table 2:** Angular Deformations of Internal Connections. Statistical comparison for the three groups before and after implant insertion. *p* values are assigned for each comparison within groups (before and after implant insertion). Significance was set as *p* < 0.05.

Angular Deformation		Sample Size	Mean	Q1	Median	Q3	*p*-Value
GROUP A	Vertex Angle Before	60	119.261	119.109	119.29	119.673	0.087
	Vertex Angle After	60	120.822	120.077	120.755	121.042	
GROUP B	Channel Angle Before	30	95.254	95.056	95.21	95.369	0.092
	Channel Angle After	30	96.829	95.68	96.48	97.83	
GROUP C	Channel Angle Before	40	91.575	77.727	93.103	93.339	0.021
	Channel Angle After	40	142.325	101.763	144.368	148.281	

**Table 3:** Wall Length Deformations of Internal Connections. Statistical comparison for the three groups before and after implant insertion. *p* values are assigned for each comparison within groups. All the groups experimented changes at the wall length. The changes were significant at group C. Significance was set as *p*-value < 0.05.

Variable		Sample Size	Mean	Difference Before vs. After	Test Statistic	*p*-Value
GROUP A Hexagon Wall Length	Before	60	1005.77 µm	−0.25	0.08683	0.9511
	After	60	1005.52 µm			
GROUP B Three-Channeled Wall Length	Before	30	2646.90 µm	−22.76	2.49081	0.08351
	After	30	2624.14 µm			
GROUP C Four-Channeled Wall Length	Before	40	655.89 µm	−186.2140	36.18923	0.00006
	After	40	469.68 µm			
